# Postdoctoral researchers' perspectives on working conditions and equal opportunities in German academia

**DOI:** 10.3389/fpsyg.2023.1217823

**Published:** 2023-09-29

**Authors:** Jacob D. Davidson, Felipe Nathan de Oliveira Lopes, Sajjad Safaei, Friederike Hillemann, Nicholas J. Russell, H. Lina Schaare

**Affiliations:** Max Planck Society PostdocNet, Munich, Germany

**Keywords:** postdoctoral researchers, academia, working conditions, equal opportunities, #IchBinHanna

## Abstract

Postdoctoral researchers (postdocs) are an essential component of the scientific workforce in German universities and research institutions and play a vital role in advancing knowledge and innovation. However, the experiences of postdocs and other early career researchers (ECRs) indicate that working conditions pose a significant challenge to the pursuit of a long-term research career in Germany—particularly for international scientists and those from marginalized groups. We examine how unstable working conditions as well as insufficient structural support for equal opportunities and diversity are significant obstacles for the career development of ECRs in German academia. We discuss these issues with the aid of an extensive survey recently conducted and published by PostdocNet, a target-group network representing the interests of postdocs across Germany's Max Planck Society. The survey drew responses from 659 postdoctoral researchers working at the Max Planck Society and represents one of the few datasets of postdoctoral researchers' perspectives in Germany. Building on these findings, we suggest actions at governmental, institutional, and individual levels to improve the working conditions of postdoctoral researchers in Germany.

## 1. Academic work in Germany is predominantly fixed- and short-term

Fixed-term employment contracts are prevalent among academic staff, especially early career researchers (ECRs), who include doctoral researchers, postdocs, and principal investigators in third-party-funded projects. In Germany, a 2021 report states that a staggering 92% of academic staff under the age of 45 who have not reached the rank of full professors are on fixed-term employment contracts (Konsortium Bundesbericht Wissenschaftlicher Nachwuchs, 2021). Multiple studies have demonstrated that the fraction employed on fixed-term contracts is significantly higher in Germany compared to other countries (we note, however, that differences in categories and nomenclature make exact quantitative comparisons difficult; e.g., a 2016 report using different data listed a fraction of 87% in Germany; Statistisches Bundesamt, 2018; Rahal et al., 2023). In comparison, approximately 60% of academic or research staff in the Netherlands are on fixed-term contracts (Rahal et al., 2023; Universiteiten van Nederland (UNL), 2023), whereas France, the United Kingdom, and Sweden have between 30 and 35% of fixed-term academic staff (Kreckel and Zimmermann, 2014; Kreckel, 2016; Haglund, 2018; Deutscher Bundestag, 2022; Higher Education Statistics Agency, 2022; Rahal et al., 2023). Moreover, the duration of German academic contracts is often short. A survey of 5,700 academics by The German Trade Union Confederation (*Der Deutsche Gewerkschaftsbund*) found that 84% of ECRs are employed on contracts lasting less than 18 months while a quarter of these scientists have already worked on four or more such contracts (Bolenius, 2020).

In Germany's non-academic sectors, fixed-term employment is strongly regulated by the act on part-time and temporary work (*Teilzeit- und Befristungsgesetz*). This law stipulates that fixed-term employment must not exceed a maximum of 2 years, after which the employee is required to transition to a permanent position if they stay within the same organization. In contrast, German academic contracts are regulated by the *Wissenschaftszeitvertragsgesetz* (WissZeitVG), which allows for a longer period of temporary employment. In its current form, the WissZeitVG allows for a maximum of 12 years of temporary employment—6 years of temporary employment before and 6 years of employment after the completion of the PhD (these time limits can be extended under certain circumstances such as parental leave or if the employment is funded by third-party sources). The WissZeitVG aims to provide scientists with adequate, but limited, time to attain the necessary qualifications for a tenured position while ensuring a continuous influx of junior researchers bringing new research concepts and ideas (Kubon, 2021; BMBF, 2023). Additionally, placing limits on fixed-term contracts was intended to alleviate the long-term employment uncertainty faced by scientists. In practice, however, the implementation of the WissZeitVG has contributed to precarious working conditions for researchers: the percentage of people employed under fixed- and short-term contracts is significantly higher than the national average, and the WissZeitVG has facilitated the exploitation of ECRs (Kubon, 2021). In addition, unlike rules in Austria and Sweden which impose time limits on postdoctoral work contracts but “reset” them when one changes universities or employers (Bundesministerium für Bildung, Wissenschaft und Forschung, Österreich (BMBWF Austria), 2021; Rahal et al., 2023), the WissZeitVG applies to all pre- and postdoctoral experience gained within the German academic system and thus sets a strict timeframe for German academic careers. Since its inception, the WissZeitVG has undergone several amendments and evaluations and its reform is currently under discussion.

Within the Max Planck Society (*Max-Planck-Gesellschaft* or MPG; a German non-university research organization dedicated to basic research), the PostdocNet represents the postdoctoral community across the 85 associated institutes. The organization is committed to providing strong support to its postdocs in advancing their personal scientific development and in reaching their personal goals for further qualification, within and outside of academia. As part of its target-group network activities, PostdocNet conducted surveys of MPG postdocs in 2019 (*n* = 623) and 2022 (*n* = 659). Both surveys confirm Germany-wide trends regarding fixed-term academic employment among ECRs (we note, however, that compared to university postdocs, MPG ECRs enjoy many advantages, such as teaching exemption and less dependence on third-party funding) (Vallier et al., 2020; Russell et al., 2023). The 2022 survey results show that 85% of postdoc respondents are on work contracts and 10% are funded through stipends or fellowships (remaining 5%: other or no response) (Russell et al., 2023). Notably, in 1976 just about 16% percent of all scientific staff at the MPG were fixed-term, by 2016 that figure had risen to 69% (Leendertz, 2020). However, non-European postdocs reported more frequently to be employed on stipends than European and German postdocs in the 2022 survey (Russell et al., 2023). Stipends have numerous disadvantages compared to work contracts, as they do not qualify as employment *per se*. For instance, stipend holders cannot obtain employer contributions to public health and unemployment insurance. The recent 2022 PostdocNet survey results (Russell et al., 2023) indicate that unequal treatment of postdocs has decreased since the 2019 survey, but still persist (e.g., the percentage of stipend or fellowship holders decreased from 13 to 10% from 2019 to 2022) (Vallier et al., 2020). Since the publication of the 2019 survey, the MPG has introduced measures that favor hiring postdocs with contracts over stipend-based employment. The organization is also working toward standardizing the initial contract length and salaries for incoming postdocs.

## 2. Working conditions affect both research and researchers

**Figure 1:**
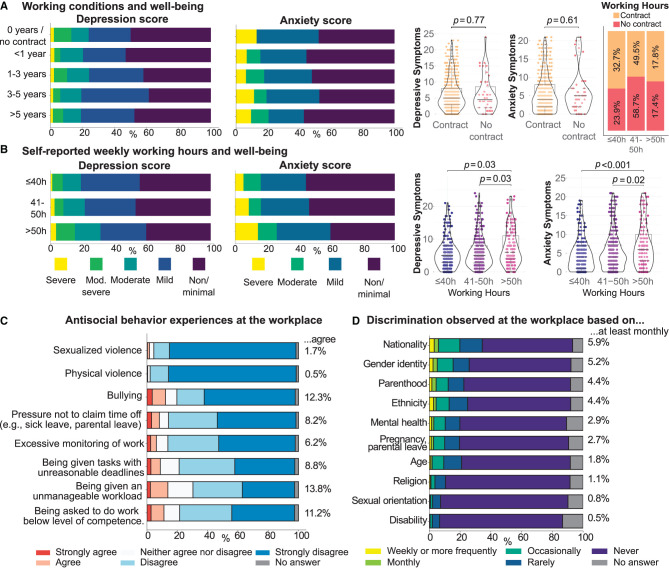
Selected results from the PostdocNet 2022 survey. Relationship of depressive symptoms measured using the Patient Health Questionnaire (PHQ-8) and anxiety symptoms measured using the Generalized Anxiety Disorder screener (GAD-7) with **(A)** working conditions and **(B)** self-reported working hours per week. **(C)** The answers of survey respondents to questions regarding workplace experiences, with stacked bar charts showing the percentage of each answer. The total percentages of agreement with each question shown to the right include both *Agree* and *Strongly agree* answers. **(D)** Answers of survey respondents to the question “During the last 12 months, how often have you observed a situation in your work environment in which one or more individuals were treated differently and/or with contempt/condescension because of the following characteristics?” Figures adapted from the PostdocNet Survey 2022 Report (Russell et al., 2023) and created in part using *ggstatsplot* (Patil, 2021).

Short-term employment opportunities and uncertainty over contract renewals make it much more difficult for scientists to plan for the future, both in their personal lives and in their research endeavors. This is exacerbated by the fact that while the majority of ECRs aspire to an academic career (~75% of surveyed postdocs in the PostdocNet 2022 survey, Russell et al., 2023), only a small percentage will eventually obtain a tenured position. Those who do not obtain permanent employment face a countdown until they are forced out of the system. This results in a highly uncertain and competitive work culture that has a strong selection bias against researchers from underrepresented groups and discourages many bright scientists from pursuing an academic career at an early stage. Unreasonably heavy workloads and poor working conditions can also adversely affect wellbeing. PostdocNet's 2022 survey results show, unsurprisingly, that work is the largest stressor for postdocs, with 73% of respondents reporting they are bothered by stress at work (Russell et al., 2023). Furthermore, surveyed postdocs who were not employed on a contract (i.e., those with stipends/fellowships) more often reported higher levels of moderate-severe depressive and anxiety symptoms than postdocs with contracts (depressive symptoms: no contract = 13% (moderately) severe, with contract = 6–10% (moderately) severe; anxiety symptoms: no contract = 13% severe, with contract = 6–11% severe). However, this relationship between working conditions and psychological wellbeing was not significant [Figure 1A; full model fit for depressive symptoms: *t*_Student_(652) = 0.29, *p* = 0.77, ĝ_Hedges_ = 0.04, CI_95%_[−0.27, 0.23], *n*_obs_ = 654; full model fit for anxiety symptoms: *t*_Student_(652) = −0.52, *p* = 0.61, ĝ_Hedges_ = −0.07, CI_95%_[−0.38, 0.24], *n*_obs_ = 654]. Approximately 67% of surveyed postdocs reported working more than 40h per week, despite the fact that contractually agreed working hours are typically 39h per week. The fraction of postdocs without a contract (i.e., with stipends/fellowships) who reported working more than 40h per week was higher than those with a contract, though not significantly [76 vs. 67%, $\chi_{\text{Pearson}}^{2}\left(1,n_{\text{obs}}=654\right)=1.15$, *p* = 0.28; Figure 1A, right]. Working more than 50h per week was reported by approximately 18% of surveyed postdocs, and was significantly associated with more severe depressive symptoms [$\chi_{\text{Kruskal-wallis}}^{2}\left(2\right)=7.21$, *p*_Holm-adj._ = 0.03, ${\hat{\epsilon }}_{\text{ordinal}}^{2}=0.01$, ${\text{CI}}_{95\%}\left[1.92\times 10^{-3},1.00\right]$, *n*_obs_ = 659; *post-hoc* comparisons using the Dunn test: ≤ 40h vs. >50h *p*_Holm-adj._ = 0.03, 41 − 50h vs. >50h *p*_Holm-adj._ = 0.03] and anxiety symptoms [$\chi_{\text{Kruskal-wallis}}^{2}\left(2\right)=11.99$, $p_{\text{Holm-adj.}}=2.49\times 10^{-3}$, ${\hat{\epsilon }}_{\text{ordinal}}^{2}=0.02$, CI_95%_[0.01, 1.00], *n*_obs_ = 659; *post-hoc* comparisons using the Dunn test: ≤ 40h vs. >50h $p_{\text{Holm-adj.}}=1.70\times 10^{-3}$, 41 − 50h vs. >50h *p*_Holm-adj._ = 0.02; Figure 1B].

Employment uncertainty not only contributes to high levels of stress but may also harm scientific progress: innovative, but also time-consuming, inter- and multidisciplinary research projects are barely possible under the time pressure of a contract coming to an end. Furthermore, projects often face a lack of continuity as experienced researchers are compelled or forced to leave the German academic system. This disrupts the research workflow and hinders the overall progress of projects (Bradler and Roller, 2023; Rahal et al., 2023). Instead of encouraging scientific innovation, the insistence on short-term contracts discourages long-term or ambitious and cutting-edge projects that challenge status-quo scientific concepts (Park et al., 2023). Projects perceived to carry risks often hold the potential for high rewards and may lead to innovative or ground-breaking outcomes. Employment uncertainty encourages researchers to propose less risky projects that can be completed within typical short-term funding cycles.

## 3. Academic diversity and equal opportunities are shaped by working conditions

In Germany, a significant portion of the scientific workforce consists of international postdocs. Due to the importance of diversity to the functioning of groups and organizations (Hong and Page, 2004; Page, 2007; Herring, 2009; Woolley et al., 2010; Freeman and Huang, 2014), the unique perspectives and expertise of researchers from diverse backgrounds are necessary to ensure a flourishing academic landscape and address skill shortages in Germany's non-academic sectors. In industry, it is acknowledged that diversity can enhance innovation and creativity (Lee, 2015; Paulus et al., 2016; Hunt et al., 2018). In academia, publications with a diverse group of authors tend to receive more citations (AlShebli et al., 2018). There is also an acknowledgment that global perspectives and a broad range of research topics are needed to ensure that scientific research remains robust to address the large-scale problems societies face in an increasingly interconnected world (Graves et al., 2022; Kozlowski et al., 2022). However, recent work has highlighted, for example, that Black, Hispanic, and Asian or Pacific Islander scientists face additional barriers within the scientific publishing system (Liu et al., 2023). Other studies and perspectives have noted how women and underrepresented groups face additional obstacles to success in Germany and the rest of the world (Hofstra et al., 2020; Cech and Waidzunas, 2021; Llorens et al., 2021; Morgan et al., 2022).

To fully understand the broad implications of how employment conditions and prospects impact diversity in academia, it is important to remember that the postdoctoral phase is not only a time for scientific and career development but often coincides with significant life events such as starting a family or taking care of aging family members. Of surveyed MPG postdocs, 28% are parents, of whom 9% became parents while working as a postdoc (Russell et al., 2023). There are more male than female parents, though not significantly [31 to 25%, $\chi_{\text{Pearson}}^{2}\left(1,n_{\text{obs}}=643\right)=2.62$, *p* = 0.11], and the number of German parents is higher than non-German parents [42 to 24%, $\chi_{\text{Pearson}}^{2}\left(1,n_{\text{obs}}=654\right)=18.37$, *p* = 1.82 × 10^−5^]; this may indicate that combining the demands of a postdoc position with those of caring for family is less attractive for women and international postdocs (Russell et al., 2023). Moreover, 75% of surveyed MPG postdocs come from outside of Germany and 50% come from outside the EU (Russell et al., 2023). This percentage of international postdocs is much higher compared to other German research institutions; for example, the Leibniz Institutes report that roughly 20% of their postdocs are non-German (Fiedler et al., 2022).

Respondents in the PostdocNet 2022 survey also reported their experiences with antisocial behavior and discrimination at work. Overall, 30% of postdocs (~200 individuals) reported having experienced some form of antisocial behavior at work (Figure 1C). The overall percentage is in alignment with the results of the Max Planck PhDnet survey, which found that 25% of doctoral researchers in the MPG have faced antisocial behavior at work, including discrimination and involvement in serious conflicts (Majev et al., 2021). Furthermore, 12% of surveyed postdocs observe discrimination at least monthly, and more than 6% of surveyed postdocs report facing discrimination at least monthly. Survey respondents most commonly attributed discrimination to factors such as nationality, gender identity, parenthood, and ethnicity (Figure 1D). Moreover, the 2022 survey showed that women reported experiencing at least monthly discrimination more than twice as often as men [9% of women to 4% of men, $\chi_{\text{Pearson}}^{2}\left(1,n_{\text{obs}}=659\right)=6.72$, *p* = 0.01]^1^ (Russell et al., 2023). We note that data on mental wellbeing and discrimination experiences were not part of the previous PostdocNet survey (Vallier et al., 2020), so we cannot compare how these trends have changed over time.

In summary, ECR's working conditions in academia do not yet provide adequate support for women scientists, researchers from marginalized groups, and all those who take on additional care work. The above mentioned disparities contribute to the gender gap in senior academic leadership positions and have the overall effect of reducing diversity in academia (Morgan et al., 2021; Zheng et al., 2022). In addition, academic working conditions have a major impact on the lives of international and non-German-speaking researchers (e.g., in terms of obtaining visas, integration in Germany, and family support). Thus, more effort must be made to include these postdocs' experiences in discussions about the reform of related laws and regulations.^2^ To gain a deeper understanding of how the laws impact researchers, as well as directions for possible change, the next section gives an overview of the legal frameworks for diversity and equal opportunities in Germany.

## 4. Legal frameworks for diversity and equal opportunities in Germany

The legal framework for diversity and equal opportunity is shaped by a number of important statutes in Germany, the most foundational being the country's Basic Law (*Grundgesetz*) (1949). There is also the General Equal Treatment Act (*Allgemeines Gleichbehandlungsgesetz* or AGG) (2006) as well as the Federal Act on Gender Equality (*Bundesgleichstellungsgesetz*) (2015). Article 3 of the *Grundgesetz* stipulates that “[a]ll people shall be equal before the law,” and in 3(2) goes further to state that “[t]he state shall promote the actual implementation of equal rights for women and men and take steps to eliminate disadvantages that now exist” (Bundesamt für Justiz, 2022). The following provision, Article 3(3), prohibits discrimination based on other characteristics. The 2006 AGG, incorporating the EU's equality directives into German law, seeks to prevent or cease discrimination on the grounds of race, ethnicity, gender, religion or belief, disability, age or sexual orientation (Federal Anti-Discrimination Agency, 2010).

While the need to address different forms of discrimination is stipulated in both the Basic Law and AGG, the wording and structure of the law [cf. Article 3(2)] give particular attention and priority to the specific disadvantages faced by women in a way that also addresses empowerment. This has enabled mandatory attention to be given to disadvantages faced by women. One example of these efforts is the 2015 Federal Act on Gender Equality (*Bundesgleichstellungsgesetz*), which has served as a legal basis for promoting an increase in the number of women in leadership roles. Another example is the requirement for public authorities, academic institutions, and private businesses to appoint a Gender Equality Officer (*Gleichstellungsbeauftragte*), who is responsible for ensuring gender equality in employment conditions and opportunities and participates in decision-making processes affecting gender equality, such as recruitment, promotion, and training (Bundesamt für Justiz, 2015).^3^

Beyond gender, other aspects of diversity, such as race, ethnicity, and sexual orientation, continue to receive insufficient attention and action in Germany. As outlined by the concept of intersectionality, it is essential to consider these factors alongside gender while formulating effective policies. Intersectionality acknowledges that multiple characteristics interact and combine to create distinct forms of inequality (Crenshaw, 1989; Leggon, 2006; Kozlowski et al., 2022).

## 5. Target-group networks and surveys provide benefit to researchers and institutions

In 2021, the German Federal Ministry of Education and Research (*Bundesministerium für Bildung und Forschung* or BMBF) released a video explaining the WissZeitVG through a fictional archetypical academic character named Hanna. Hanna was used to describe the alleged benefits of the law, such as the notion that a fast turnover of scientists prevents “clogging the system” and drives innovation. Many ECRs felt the video was not reflective of their real-life situation, with many highlighting the pressure associated with the precariousness of short-term contracts and the difficulties of following an academic career path during a time when many would want to start or care for family, in addition to the competitiveness of modern academia. In response to this video, a wave of protests on the WissZeitVG using the hashtags #IchBinHanna and #IchBinReyhan (“I am Hanna” and “I am Reyhan,” respectively) sparked a series of heated public debates between thousands of researchers and scientific stakeholders on the working conditions in German academia and the structural barriers faced by academics from marginalized backgrounds (Bahr et al., 2021, 2022; Dirnagl, 2022). These discussions have played an important role in motivating Germany-based researchers at all stages of their careers to form interest groups, grassroots initiatives, and target-group networks that facilitate public discourse on the importance of good working conditions as an integral part of a productive research environment (Bahr et al., 2022; Rahal et al., 2023).

Large academic institutions struggle to comprehend the personal concerns and challenges faced by each type of employee. Therefore, target-group networks such as PostdocNet offer a unique service to their institutions by providing a window into the worlds of their employees. Specifically, target-group networks can provide institutions with survey data to enable more streamlined assistance, for instance to address problems whose solutions would most benefit postdocs. Surveys can therefore be a powerful tool to gain insights about employees' work experiences and to identify areas of improvement, which can inform policy.

Making equity, diversity, and inclusion (EDI) issues visible is crucial to addressing the negative effects of structural biases on individuals who encounter discrimination. Evidence-based policy can be an informed way to address issues related to EDI. In the UK higher education sector, such policy mechanisms have been established by the Athena Swan gender equality initiative (Barnard, 2017) and the Race Equality Charter. Although in Germany data protection measures are strongly regulated, the importance of EDI data has recently come into focus (Aikins et al., 2021; Foroutan et al., 2022; Meyer et al., 2022; Boytchev, 2023).

We note that while surveys are a powerful tool for improvement, it is also important to mention the possible biases that can introduce incomplete or skewed understandings of people's experiences. Participants with negative experiences may be more likely to respond to surveys, while those with more positive experiences may provide inaccurate information due to memory biases. Additionally, respondents may provide socially desirable responses even when anonymously recorded. Here, target-group network surveys can supplement organizational employee surveys, as employees may feel less pressure to provide socially desirable responses. Survey responses are inherently subjective and based on participants' current perceptions and interpretations. Therefore, most survey data can reveal correlations but may not determine causality. Yet, comprehensive and longitudinal surveys can provide long-term insight into institutional culture and management practices affecting the work environment and employees' wellbeing. For this reason, PostdocNet intends to conduct regular surveys among MPG postdocs to follow the development of their needs and their work satisfaction over longer time periods. The survey method is also subject to biases from the authors, which can influence the design and analysis. In this respect, the diverse perspectives of survey designers and respondents both play a crucial role. Overall, surveys must be carefully designed and conducted; when done so, and when considered in conjunction with additional qualitative or quantitative data, target-group surveys can provide accurate and comprehensive insights on directions for improvements.

## 6. Future directions and suggested actions

Despite the crucial role postdocs play in conducting research, driving innovation, and training students (Feldon et al., 2019), the current academic system does not prioritize creating working conditions that foster good research practices and inclusivity (Rahal et al., 2023). The PostdocNet's survey results showed that, among other factors, working conditions, wellbeing and anti-discrimination measures should be a primary concern for research institutions (Figure 1) (Russell et al., 2023). Recently, the #IchBinHanna and #IchBinReyhan campaigns in Germany have been instrumental in highlighting the need for better working conditions and career development opportunities for academics (especially those at German universities), regardless of their background (Bahr et al., 2021, 2022; Rauscher, 2023).

**Figure 2:**
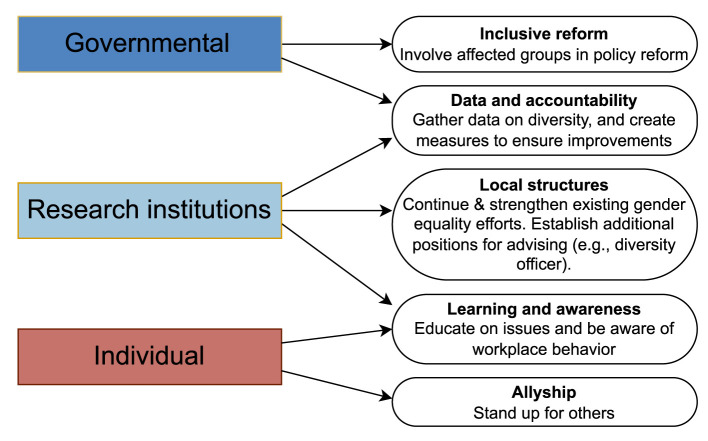
Different levels of actions. Structural changes can be at the levels of larger-scale governmental changes as well as changes at individual research institutions; some needed steps include inclusive reform, collecting data and establishing accountability measures, and creating and strengthening local structures. In addition to these, individuals can take action to be aware of issues and act as allies.

Improving laws and institutional policies is a complex process. Although there is no fast-track solution to the issues we have discussed in this paper, we can nonetheless highlight important steps and actions that may pave the way for effective change. In particular, we emphasize the need for structural changes that facilitate good working conditions and experiences for postdocs. Structural changes can be considered on the governmental and institutional (i.e., research institution) levels. In addition to structural changes, individuals can also take action. Figure 2 summarizes actions and steps that can be taken at each level in order to work toward the goals of increasing diversity in German academia and providing working conditions that enable excellent and innovative science while fostering a sustainable work atmosphere.

At the governmental level, we stress the importance of involving all stakeholders, including grassroots initiatives or target-group networks representing postdocs and international researchers, when reforming and modifying existing laws and regulations. Complementary data collection on the demographics and experiences of researchers from organizations and grassroots initiatives is a key step to monitor the effects that regulations and measures have. One can also draw inspiration for reform from similar efforts in other countries, such as the NSF Advance program in the US and the Athena Swan program and Race Equality Charter in the UK (Rosser et al., 2019; Bhopal and Henderson, 2021; Morimoto, 2022).

Improving working conditions also requires an intersectional approach to diversity, taking into account dimensions of differences and inequalities in addition to gender. It is important to note that women continue to face unique challenges in academia (Nielsen et al., 2017; Llorens et al., 2021; Ross et al., 2022); as a result, existing efforts to promote gender equality should not be replaced with broader diversity initiatives. Rather, they should be strengthened and supplemented.

While the most significant and long-lasting changes will result from structural changes at the governmental and institutional levels, individual actions can also positively affect working environments for others. These actions include learning about issues and educating oneself, as well as being aware of one's own and others' actions in the workplace. Individuals, especially those in privileged positions, can act as allies for underrepresented groups in science by ensuring that the voices of those groups are heard and by standing up against discrimination or inappropriate behavior when observed (Williams et al., 2023).

These future directions are overall part of a multifaceted approach that involves both structural and individual changes. By actively engaging in this process and prioritizing the wellbeing and success of all members of the academic community, we can create a more productive and innovative environment for research and education in Germany and beyond.

## Reflective statement

We, the authors of this perspective piece, have been involved in parts in the creation of the PostdocNet 2022 survey, and we are all postdoctoral researchers of the MPG ourselves. Since the first submission of this manuscript, HS has started to also work part-time at the MPG General Administration in the Career Development department, focusing on the postdoc phase. We acknowledge that our own biases may have influenced the survey design and analysis of the results. Our own experiences, perspectives, and assumptions could have inadvertently shaped how questions were posed, and how responses were interpreted. We made conscious efforts to address potential biases by ensuring inclusivity in our language and employing validated scales, and by engaging in iterative discussions among a diverse team of researchers, including those with backgrounds in psychology and anthropology and experience with survey design, while planning the survey and during the writing of this manuscript.

## Data availability statement

The data analyzed in this study is subject to the following licenses/restrictions: data from the PostdocNet's surveys have been referenced in this article. The full reports of the surveys are publicly available. The raw data, however, are not publicly available to ensure anonymity of the participants. Requests to access these datasets should be directed to survey@postdocnet.mpg.de.

## Author contributions

All authors contributed to the article and approved the submitted version.

## Footnotes

^1^Thirteen percent of surveyed individuals who have a different gender identity or preferred not to answer gender-related questions have experienced antisocial behavior, but the sample size was too low to be included in further analyses.

^2^ https://www.postdocnet.mpg.de/148504/Comments-on-WissZeitVG-July2023

^3^Inclusion Officers provide a comparable organizational structure for promoting inclusion of individuals with disabilities.

## References

[B1] Aikins M. A. Bremberger T. Aikins J. K. Gyamerah D. Yıldırım-Caliman D. (2021). Afrozensus 2020: Perspektiven, Anti-Schwarze Rassismuserfahrungen und Engagement Schwarzer, afrikanischer und afrodiasporischer Menschen in Deutschland, Berlin. Available online at: www.afrozensus.de

[B2] AlShebli B. K. Rahwan T. Woon W. L. (2018). The preeminence of ethnic diversity in scientific collaboration. Nat. Commun. 9, 5163. 10.1038/s41467-018-07634-8 30514841 PMC6279741

[B3] Bahr A. Blume C. Eichhorn K. Kubon S. (2021). With #IchBinHanna, German academia protests against a law that forces researchers out. Nat. Hum. Behav. 5, 1114–1115. 10.1038/s41562-021-01178-6 34341553

[B4] Bahr A. Eichhorn K. Kubon S. (2022). #IchBinHanna: Prekare Wissenschaft in Deutschland. Berlin: Suhrkamp Verlag.

[B5] Barnard S. (2017). “The Athena SWAN charter: promoting commitment to gender equality in higher education institutions in the UK,” in Gendered Success in Higher Education: Global Perspectives, eds K. White and P. O'Connor (London: Palgrave Macmillan UK), 155–174.32051310

[B6] Bhopal K. Henderson H. (2021). Competing inequalities: gender versus race in higher education institutions in the UK. Educ. Rev. 73, 153–169. 10.1080/00131911.2019.1642305

[B7] BMBF (2023). Wissenschaftszeitvertragsgesetz - BMBF.

[B8] Bolenius S. (2020). DGB-Hochschulreport - Ausgewählte Ergebnisse im Überblick. Available online at: https://www.dgb.de/themen/++co++81f5d08e-25c1-11eb-85fe-001a4a160123 (accessed June 27, 2023).

[B9] Boytchev H. (2023). Diversity in German science: researchers push for missing ethnicity data. Nature 616, 22–24. 10.1038/d41586-023-00955-9 37020000

[B10] Bradler S. Roller C. (2023). Befristung und gute wissenschaftliche praxis. Biologie in unserer Zeit 53, 12–15. 10.11576/biuz-6206

[B11] Bundesamt für Justiz (2015). Gesetz fur die Gleichstellung von Frauen und Männern in der Bundesverwaltung und in den Gerichten des Bundes. Available online at: https://www.gesetze-im-internet.de/bgleig_2015/BJNR064300015.html (accessed May 5, 2023).

[B12] Bundesamt für Justiz (2022). Basic Law for the Federal Republic of Germany (English Translation). Available online at: https://www.gesetze-im-internet.de/englisch_gg/englisch_gg.html#p0026 (accessed May 5, 2023).

[B13] Bundesministerium für Bildung Wissenschaft und Forschung, Österreich (BMBWF Austria). (2021). UG-Novelle 2021: Die wichtigsten Fragen und Antworten fur Studierende. Available online at: https://www.bmbwf.gv.at/Themen/HS-Uni/Hochschulsystem/Gesetzliche-Grundlagen/UG-Novelle-2021-faq/Fragen-und-Antworten-f?r-Universit?ts–und-Hochschulangeh?rige.html (accessed July 26, 2023).

[B14] Cech E. A. Waidzunas T. J. (2021). Systemic inequalities for LGBTQ professionals in STEM. Sci. Adv. 7, eabe0933. 10.1126/sciadv.abe0933 33523910 PMC7810386

[B15] Crenshaw K. (1989). Demarginalizing the Intersection of Race and Sex: A Black Feminist Critique of Antidiscrimination Doctrine, Feminist Theory and Antiracist Politics. University of Chicago Legal Forum.

[B16] Deutscher Bundestag (2022). Zu befristeten Arbeitsverhältnissen in der Wissenschaft und Innovation - Innovation durch Fluktuation. Technical report, Deutscher Bundestag.

[B17] Dirnagl U. (2022). #IchbinHannah and the fight for permanent jobs for postdocs. EMBO Rep. 23, e54623. 10.15252/embr.202254623 35099836 PMC8892265

[B18] Federal Anti-Discrimination Agency (2010). Guide to the General Equal Treatment Act. Explanations and Examples. MKL Druck GmbH & Co.KG, Berlin.

[B19] Feldon D. F. Litson K. Jeong S. Blaney J. M. Kang J. Miller C.. (2019). Postdocs; lab engagement predicts trajectories of PhD students' skill development. Proc. Natl. Acad. Sci. U.S.A. 116, 20910–20916. 10.1073/pnas.1912488116 31570599 PMC6800364

[B20] Fiedler D. Losch T. Heinz G. Heck T. Diez Diaz V. Repke L. (2022). Who are Leibniz Postdocs and What Is It Like to Work at a Leibniz Institute? Report of the First Leibniz Postdoc Survey 2020. Available online at: https://nbn-resolving.org/urn:nbn:de:0168-ssoar-83394-4

[B21] Foroutan N. Ha N. Kalter F. Shooman Y. Sinanoglu C. (2022). Rassistische Realitäten: Wie setzt sich Deutschland mit Rassismus auseinander? Deutsches Zentrum fur Integrations-und Migrationsforschung DeZIM e.V.

[B22] Freeman R. B. Huang W. (2014). Collaboration: strength in diversity. Nature 513, 305–305. 10.1038/513305a 25230634

[B23] Graves J. L. Kearney M. Barabino G. Malcom S. (2022). Inequality in science and the case for a new agenda. Proc. Natl. Acad. Sci. U.S.A. 119, e2117831119. 10.1073/pnas.2117831119 35210356 PMC8915968

[B24] Haglund A. (2018). Tidsbegrensade Anstellningar Bland Hogskolans Forskande och Undervisande Personal. Technical report, Universitetskanslersambetet.

[B25] Herring C. (2009). Does diversity pay?: race, gender, and the business case for diversity. Am. Sociol. Rev. 74, 208–224. 10.1177/00031224090740020334259846

[B26] Higher Education Statistics Agency (2022). Higher Education Staff Statistics: UK, 2020/21 | HESA.

[B27] Hofstra B. Kulkarni V. V. Galvez S. M.-N. He B. Jurafsky D. McFarland D. A. (2020). The diversity–innovation paradox in science. Proc. Natl. Acad. Sci. U.S.A. 117, 9284–9291. 10.1073/pnas.1915378117 32291335 PMC7196824

[B28] Hong L. Page S. E. (2004). Groups of diverse problem solvers can outperform groups of high-ability problem solvers. Proc. Natl. Acad. Sci. U.S.A. 101, 16385–16389. 10.1073/pnas.0403723101 15534225 PMC528939

[B29] Hunt V. Prince S. Dixon-Fyle S. Yee L. (2018). Delivering through Diversity. Report, McKinsey & Company.

[B30] Konsortium Bundesbericht Wissenschaftlicher Nachwuchs (2021). Bundesbericht Wissenschaftlicher Nachwuchs 2021. wbv Media, DE.

[B31] Kozlowski D. Lariviore V. Sugimoto C. R. Monroe-White T. (2022). Intersectional inequalities in science. Proc. Natl. Acad. Sci. U.S.A. 119, e2113067119. 10.1073/pnas.2113067119 34983876 PMC8764684

[B32] Kreckel R. (2016). Zur Lage des wissenschaftlichen Nachwuchses an Universitäten: Deutschland im Vergleich mit Frankreich, England, den USA und Österreich. Beitrage zur Hochschulforschung 38, 12–40. Available online at: https://www.bzh.bayern.de/fileadmin/news_import/1-2-2016-Kreckel.pdf

[B33] Kreckel R. Zimmermann K. (2014). Hasard oder Laufbahn.Akademische Karrierestrukturen im internationalen Vergleich. HoF.

[B34] Kubon S. (2021). “Frist first: Über die entstehung des wissenschafts-zeitvertragsgesetzes und die begriffe innovation, fluktuation und qualifikation als ideologische grundlagen und dogmen,” in #95vsWissZeitVG. Prekäre Arbeit in der deutschen Wissenschaft (Marburg: Büchner Verlag), 12–32.

[B35] Lee N. (2015). Migrant and ethnic diversity, cities and innovation: firm effects or city effects? J. Econ. Geogr. 15, 769–796. 10.1093/jeg/lbu032

[B36] Leendertz A. (2020). Wissenschaftler auf zeit: Die durchsetzung der personalpolitik der befristung in Der Max-Planck-Gesellschaft seit den 1970er-jahren. *MPIfG Discuss. Pap*. 20. Available online at: https://hdl.handle.net/21.11116/0000-0007-9E71-8

[B37] Leggon C. B. (2006). Women in science: racial and ethnic differences and the differences they make. J. Technol. Transfer 31, 325–333. 10.1007/s10961-006-7204-2

[B38] Liu F. Rahwan T. AlShebli B. (2023). Non-White scientists appear on fewer editorial boards, spend more time under review, and receive fewer citations. Proc. Natl. Acad. Sci. U.S.A. 120, e2215324120. 10.1073/pnas.2215324120 36940343 PMC10068789

[B39] Llorens A. Tzovara A. Bellier L. Bhaya-Grossman I. Bidet-Caulet A. Chang W. K.. (2021). Gender bias in academia: a lifetime problem that needs solutions. Neuron 109, 2047–2074. 10.1016/j.neuron.2021.06.002 34237278 PMC8553227

[B40] Majev P.-G. Vieira R. M. Carollo A. Liu H. Stutz D. Fahrenwaldt A.. (2021). PhDnet Report 2020. UNAS.

[B41] Meyer J. Strauß S. Hinz T. (2022). Die Studierendenbefragung in Deutschland: Fokusanalysen zu Diskriminierungserfahrungen an Hochschulen. Deutsches Zentrum fur Hochschul- und Wissenschaftsforschung (DZHW).

[B42] Morgan A. C. LaBerge N. Larremore D. B. Galesic M. Brand J. E. Clauset A. (2022). Socioeconomic roots of academic faculty. Nat. Hum. Behav. 6, 1625–1633. 10.1038/s41562-022-01425-4 36038774 PMC9755046

[B43] Morgan A. C. Way S. F. Hoefer M. J. D. Larremore D. B. Galesic M. Clauset A. (2021). The unequal impact of parenthood in academia. Sci. Adv. 7, eabd1996. 10.1126/sciadv.abd1996 33627417 PMC7904257

[B44] Morimoto S. A. (2022). The social science of institutional transformation: intersectional change in the academy. Front. Sociol. 7, 824497. 10.3389/fsoc.2022.824497 35495571 PMC9049015

[B45] Nielsen M. W. Alegria S. Borjeson L. Etzkowitz H. Falk-Krzesinski H. J. Joshi A.. (2017). Opinion: gender diversity leads to better science. Proc. Natl. Acad. Sci. U.S.A. 114, 1740–1742. 10.1073/pnas.1700616114 28228604 PMC5338420

[B46] Page S. E. (2007). Making the difference: applying a logic of diversity. Acad. Manage. Perspect. 21, 6–20. 10.5465/amp.2007.27895335

[B47] Park M. Leahey E. Funk R. J. (2023). Papers and patents are becoming less disruptive over time. Nature 613, 138–144. 10.1038/s41586-022-05543-x 36600070

[B48] Patil I. (2021). Visualizations with statistical details: the 'ggstatsplot' approach. J. Open Source Softw. 6, 3167. 10.21105/joss.03167

[B49] Paulus P. B. van der Zee K. I. Kenworthy J. (2016). “Cultural diversity and team creativity,” in The Palgrave Handbook of Creativity and Culture Research, ed V. P. Glaveanu (London: Palgrave Macmillan UK), 57–76.

[B50] Rahal R.-M. Fiedler S. Adetula A. Berntsson R. P.-A. Dirnagl U. Feld G. B.. (2023). Quality research needs good working conditions. Nat. Hum. Behav. 7, 164–167. 10.1038/s41562-022-01508-2 36755134

[B51] Rauscher J. (2023). Fur eine umfassende Reform des Wissenschaftszeitvertragsgesetzes. Helmholtz Blogs.

[B52] Ross M. B. Glennon B. M. Murciano-Goroff R. Berkes E. G. Weinberg B. A. Lane J. I. (2022). Women are credited less in science than men. Nature 608, 135–145. 10.1038/s41586-022-04966-w 35732238 PMC9352587

[B53] Rosser S. V. Barnard S. Carnes M. Munir F. (2019). Athena SWAN and ADVANCE: effectiveness and lessons learned. Lancet 393, 604–608. 10.1016/S0140-6736(18)33213-6 30739697

[B54] Russell N. J. Schaare H. L. Bellon Lara B. Dang Y. Feldmeier-Krause A. Meemken M.-T.. (2023). Max Planck PostdocNet Survey Report 2022. Max Planck Society PostdocNet. 10.17617/2.3507886

[B55] Statistisches Bundesamt (2018). Hochschulen auf einen Blick – Ausgabe 2018. Technical report, Statistisches Bundesamt (Destatis).

[B56] Universiteiten van Nederland (UNL) (2023). Wetenschappelijk Onderwijs PersoneelsInformatie (WOPI). Available online at: https://www.universiteitenvannederland.nl/f_c_personeel_downloads.html (accessed July 26, 2023).

[B57] Vallier M. Mueller M. Alcami P. Bellucci G. Grange M. Lu Y.. (2020). Demographics and employment of Max-Planck Society's postdocs. bioRxiv. 10.1101/2020.11.27.399733

[B58] Williams M. T. Faber S. Nepton A. Ching T. H. W. (2023). Racial justice allyship requires civil courage: a behavioral prescription for moral growth and change. Am. Psychol. 78, 1–19. 10.1037/amp0000940 35143235

[B59] Woolley A. W. Chabris C. F. Pentland A. Hashmi N. Malone T. W. (2010). Evidence for a collective intelligence factor in the performance of human groups. Science 330, 686–688. 10.1126/science.1193147 20929725

[B60] Zheng X. Yuan H. Ni C. (2022). How parenthood contributes to gender gaps in academia. eLife 11, e78909. 10.7554/eLife.78909 35822694 PMC9299837

